# 1-(4-Chloro­benzo­yl)-3-(2,4,6-trichloro­phen­yl)thio­urea hemihydrate

**DOI:** 10.1107/S1600536808041251

**Published:** 2008-12-17

**Authors:** M. Khawar Rauf, Michael Bolte, Amin Badshah

**Affiliations:** a Department of Chemistry, Quaid-i-Azam University Islamabad, 45320-Pakistan; b Institut für Anorganische Chemie, J. W. Goethe-Universität Frankfurt, Max-von-Laue-Str. 7, 60438 Frankfurt/Main, Germany

## Abstract

The asymmetric unit of the title compound, C_14_H_8_Cl_4_N_2_OS·0.5H_2_O, contains two independent mol­ecules with different conformations with respect to the aromatic ring planes, and one water mol­ecule. The bond lengths and angles are typical of thio­urea compounds of this class. The mol­ecule exists in the solid state in its thione form with typical thio­urea C—S and C—O bonds lengths, as well as shortened C—N bonds. The dihedral angles between the two aromatic planes are 66.93 (8) and 60.44 (9)° in the two independent mol­ecules. An intra­molecular N—H⋯O hydrogen bond stabilizes the mol­ecular conformation and the crystal packing is characterized by N—H⋯O, O—H⋯S and O—H⋯Cl hydrogen bonds.

## Related literature

For background and related structures, see: Khawar Rauf *et al.* (2006*a* ▶,*b* ▶,*c* ▶,*d* ▶). For a description of the Cambridge Structural Database, see: Allen (2002 ▶).
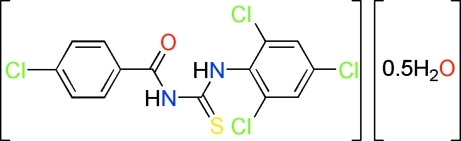


## Experimental

### 

#### Crystal data

C_14_H_8_Cl_4_N_2_OS·0.5H_2_O
*M* *_r_* = 403.09
Monoclinic, 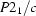

*a* = 16.1428 (9) Å
*b* = 13.7340 (7) Å
*c* = 16.2850 (9) Å
β = 112.216 (4)°
*V* = 3342.4 (3) Å^3^
*Z* = 8
Mo *K*α radiation
μ = 0.84 mm^−1^
*T* = 173 (2) K
0.38 × 0.37 × 0.35 mm

#### Data collection

STOE IPDS II two-circle-diffractometer
Absorption correction: multi-scan (*MULABS*; Spek, 2003 ▶; Blessing, 1995 ▶) *T*_min_ = 0.741, *T*_max_ = 0.758
23391 measured reflections
7172 independent reflections
5964 reflections with *I* > 2σ(*I*)
*R*_int_ = 0.068

#### Refinement

*R*[*F* ^2^ > 2σ(*F* ^2^)] = 0.045
*wR*(*F* ^2^) = 0.125
*S* = 1.02
7172 reflections
428 parameters
7 restraints
H atoms treated by a mixture of independent and constrained refinement
Δρ_max_ = 0.94 e Å^−3^
Δρ_min_ = −0.60 e Å^−3^

### 

Data collection: *X-AREA* (Stoe & Cie, 2001 ▶); cell refinement: *X-AREA*; data reduction: *X-AREA*; program(s) used to solve structure: *SHELXS97* (Sheldrick, 2008 ▶); program(s) used to refine structure: *SHELXL97* (Sheldrick, 2008 ▶); molecular graphics: *XP* in *SHELXTL-Plus* (Sheldrick, 2008 ▶); software used to prepare material for publication: *SHELXL97*.

## Supplementary Material

Crystal structure: contains datablocks I, global. DOI: 10.1107/S1600536808041251/si2141sup1.cif

Structure factors: contains datablocks I. DOI: 10.1107/S1600536808041251/si2141Isup2.hkl

Additional supplementary materials:  crystallographic information; 3D view; checkCIF report

## Figures and Tables

**Table 1:** Hydrogen-bond geometry (Å, °)

*D*—H⋯*A*	*D*—H	H⋯*A*	*D*⋯*A*	*D*—H⋯*A*
N1—H1⋯O1*W*	0.866 (10)	2.211 (17)	2.997 (3)	151 (3)
N2—H2⋯O1	0.873 (10)	1.97 (2)	2.627 (2)	131 (3)
N2—H2⋯O1*A*^i^	0.873 (10)	2.26 (2)	2.931 (3)	133 (2)
N1*A*—H1*A*⋯O1*W*	0.874 (10)	1.964 (13)	2.816 (3)	164 (3)
N2*A*—H2*A*⋯O1*A*	0.877 (10)	1.98 (3)	2.637 (3)	130 (3)
N2*A*—H2*A*⋯O1^ii^	0.877 (10)	2.31 (2)	3.001 (3)	136 (3)
O1*W*—H1*WA*⋯S1*A*	0.855 (10)	2.67 (3)	3.215 (2)	123 (3)
O1*W*—H1*WA*⋯Cl3^iii^	0.855 (10)	2.84 (3)	3.388 (2)	123 (3)
O1*W*—H1*WB*⋯S1	0.855 (10)	2.36 (2)	3.091 (2)	144 (3)

Symmetry codes: (i) 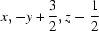; (ii) 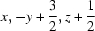; (iii) 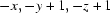.

**Fig. 1.**
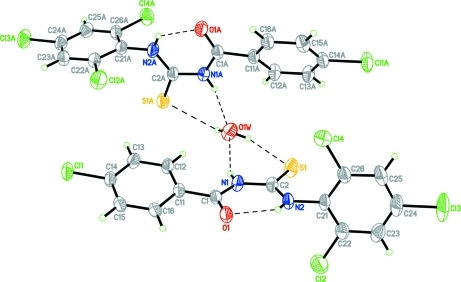
Molecular structure of (I) showing the atom numbering scheme. Intra- and intermolecular hydrogen bonds are shown as dash lines. Displacement ellipsoids are drawn at the 50% probability level.

**Fig. 2.**
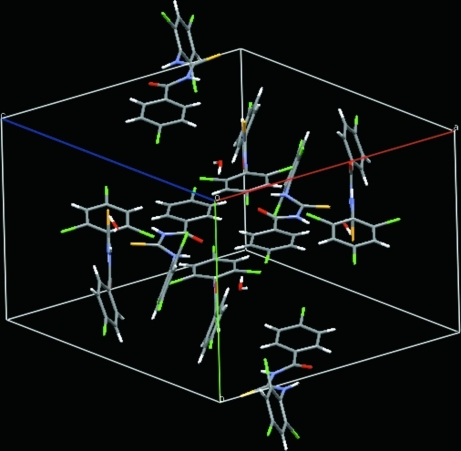
Packing diagram of (I).

## supplementary crystallographic information

### Comment

The background to this study has been set out in our previous work on the structural chemistry of *N*,*N'*-disubstituted thioureas (Khawar Rauf *et al.*, 2006*a*, 2006*b*, 2006*c*, 2006*d*). Herein, as a continuation of these studies, the structure of the title compound (I) is described. A depiction of the two independent molecules is given in Fig. 1.The water molecule links the molecules through O_W_—H···S and N—H···O_W_ hydrogen bonds. Bond lengths and angles, can be regarded as typical for *N*,*N'*-disubstituted thiourea compounds as found in the Cambridge Structural Database v5.28 (Allen, 2002; Khawar Rauf *et al.*,2006*b*).The molecule exists in the thione form with typical thiourea C—S and C—O bonds, as well as shortened C—N bond lengths.The thiocarbonyl and carbonyl groups are almost coplanar. An intramolecular N—H···O hydrogen bond is present, forming a six membered ring commonly observed in this class of compounds (Khawar Rauf *et al.*, 2006*a*). The the crystal packing is stabilized by intermolecular N—H···O, O—H···S and O—H···Cl hydrogen bonds (Table 1).

### Experimental

Freshly prepared 4-chlorobenzoyl isothiocyanate (2.0 g, 10 mmol) was stirred in acetone (40 ml) for 15 minutes. Neat 3,5-dichloroaniline (1.62 g, 10 mmol) was then added and the resulting mixture was stirred for 1 h. The reaction mixture was then poured into acidified (pH 4) water and stirred well. The solid product was separated and washed with deionized water and purified by recrystallization from methanol/ 1,1-dichloromethane (1:10 *v/v*) to give fine crystals of (I), with an overall yield of 85%. Full spectroscopic and physical characterization will be reported elsewhere.

### Refinement

Hydrogen atoms bonded to C were included in calculated positions and refined as riding on their parent C atom with C—H = 0.95 Å *U*_iso_(H) = 1.2*U*_eq_(C). The H atoms bonded to N were refined with the N—H distances restrained to 0.88 (1) Å. The water H atoms were refined with the O—H distances restrained to 0.83 (1)Å and the H···H distances restrained to 1.40 (5)Å and with *U*_iso_(H) = 1.2*U*_eq_(O).

### Crystal data

**Table:**

C_14_H_8_Cl_4_N_2_OS·0.5H_2_O	*F*(000) = 1624
*M**_r_* = 403.09	*D*_x_ = 1.602 Mg m^−^^3^
Monoclinic, *P*2_1_/*c*	Mo *K*α radiation, λ = 0.71073 Å
Hall symbol: -P 2ybc	Cell parameters from 21741 reflections
*a* = 16.1428 (9) Å	θ = 2.2–27.1°
*b* = 13.7340 (7) Å	µ = 0.84 mm^−^^1^
*c* = 16.2850 (9) Å	*T* = 173 K
β = 112.216 (4)°	Block, colourless
*V* = 3342.4 (3) Å^3^	0.38 × 0.37 × 0.35 mm
*Z* = 8	

### Data collection

**Table:**

STOE IPDS II two-circle-diffractometer	7172 independent reflections
Radiation source: fine-focus sealed tube	5964 reflections with *I* > 2σ(*I*)
graphite	*R*_int_ = 0.068
ω scans	θ_max_ = 26.9°, θ_min_ = 2.1°
Absorption correction: multi-scan (*MULABS*; Spek, 2003; Blessing, 1995)	*h* = −17→20
*T*_min_ = 0.741, *T*_max_ = 0.758	*k* = −17→17
23391 measured reflections	*l* = −20→20

### Refinement

**Table:**

Refinement on *F*^2^	Primary atom site location: structure-invariant direct methods
Least-squares matrix: full	Secondary atom site location: difference Fourier map
*R*[*F*^2^ > 2σ(*F*^2^)] = 0.045	Hydrogen site location: inferred from neighbouring sites
*wR*(*F*^2^) = 0.125	H atoms treated by a mixture of independent and constrained refinement
*S* = 1.02	*w* = 1/[σ^2^(*F*_o_^2^) + (0.0647*P*)^2^ + 1.5427*P*] where *P* = (*F*_o_^2^ + 2*F*_c_^2^)/3
7172 reflections	(Δ/σ)_max_ = 0.001
428 parameters	Δρ_max_ = 0.94 e Å^−^^3^
7 restraints	Δρ_min_ = −0.60 e Å^−^^3^

### Special details

**Table:**

Geometry. All e.s.d.'s (except the e.s.d. in the dihedral angle between two l.s. planes) are estimated using the full covariance matrix. The cell e.s.d.'s are taken into account individually in the estimation of e.s.d.'s in distances, angles and torsion angles; correlations between e.s.d.'s in cell parameters are only used when they are defined by crystal symmetry. An approximate (isotropic) treatment of cell e.s.d.'s is used for estimating e.s.d.'s involving l.s. planes.
Refinement. Refinement of *F*^2^ against ALL reflections. The weighted *R*-factor *wR* and goodness of fit *S* are based on *F*^2^, conventional *R*-factors *R* are based on *F*, with *F* set to zero for negative *F*^2^. The threshold expression of *F*^2^ > σ(*F*^2^) is used only for calculating *R*-factors(gt) *etc*. and is not relevant to the choice of reflections for refinement. *R*-factors based on *F*^2^ are statistically about twice as large as those based on *F*, and *R*- factors based on ALL data will be even larger.

### Fractional atomic coordinates and isotropic or equivalent isotropic displacement parameters (Å^2^)

**Table:**

	*x*	*y*	*z*	*U*_iso_*/*U*_eq_	
C1	0.37638 (14)	0.52532 (16)	0.57504 (15)	0.0302 (5)	
O1	0.38255 (10)	0.53995 (13)	0.50312 (11)	0.0357 (4)	
N1	0.29511 (12)	0.50614 (15)	0.58141 (13)	0.0324 (4)	
H1	0.2955 (19)	0.497 (2)	0.6343 (10)	0.041 (8)*	
C2	0.21054 (14)	0.50210 (17)	0.51379 (15)	0.0325 (5)	
N2	0.20766 (12)	0.52155 (15)	0.43208 (13)	0.0332 (4)	
H2	0.2559 (13)	0.537 (2)	0.4227 (19)	0.045 (8)*	
S1	0.12057 (4)	0.47441 (6)	0.53583 (4)	0.04717 (18)	
Cl1	0.71014 (4)	0.55572 (5)	0.90337 (4)	0.04341 (16)	
Cl2	0.15939 (5)	0.34487 (6)	0.31698 (6)	0.0673 (2)	
Cl3	−0.13692 (5)	0.54231 (9)	0.12131 (5)	0.0773 (3)	
Cl4	0.11099 (5)	0.71115 (5)	0.40822 (5)	0.05120 (18)	
C11	0.45662 (14)	0.52864 (16)	0.65923 (15)	0.0295 (4)	
C12	0.45243 (15)	0.53432 (17)	0.74359 (15)	0.0333 (5)	
H12	0.3960	0.5332	0.7492	0.040*	
C13	0.53058 (16)	0.54156 (18)	0.81928 (15)	0.0355 (5)	
H13	0.5278	0.5454	0.8764	0.043*	
C14	0.61249 (15)	0.54311 (17)	0.80970 (15)	0.0337 (5)	
C15	0.61818 (15)	0.53658 (18)	0.72711 (16)	0.0359 (5)	
H15	0.6748	0.5374	0.7219	0.043*	
C16	0.54017 (15)	0.52882 (17)	0.65206 (15)	0.0332 (5)	
H16	0.5436	0.5236	0.5953	0.040*	
C21	0.12477 (14)	0.52657 (18)	0.35815 (15)	0.0335 (5)	
C22	0.09467 (16)	0.44876 (19)	0.29937 (18)	0.0406 (6)	
C23	0.01410 (17)	0.4524 (2)	0.22662 (18)	0.0473 (7)	
H23	−0.0059	0.3986	0.1874	0.057*	
C24	−0.03568 (16)	0.5362 (2)	0.21313 (18)	0.0492 (7)	
C25	−0.00886 (16)	0.6165 (2)	0.26850 (18)	0.0461 (6)	
H25	−0.0447	0.6735	0.2577	0.055*	
C26	0.07260 (15)	0.61070 (19)	0.34072 (16)	0.0371 (5)	
C1A	0.24641 (14)	0.78726 (17)	0.78708 (15)	0.0314 (5)	
O1A	0.28145 (10)	0.86354 (12)	0.82401 (11)	0.0359 (4)	
N1A	0.27773 (12)	0.69679 (15)	0.82106 (13)	0.0328 (4)	
H1A	0.2541 (18)	0.6458 (14)	0.7884 (16)	0.044 (8)*	
C2A	0.34638 (14)	0.67443 (17)	0.90195 (15)	0.0313 (5)	
N2A	0.38814 (13)	0.75109 (15)	0.95146 (13)	0.0344 (4)	
H2A	0.374 (2)	0.8114 (10)	0.934 (2)	0.052 (9)*	
S1A	0.37231 (4)	0.55898 (5)	0.93165 (4)	0.03769 (15)	
Cl1A	−0.07853 (4)	0.81919 (6)	0.45000 (5)	0.05347 (19)	
Cl2A	0.57143 (5)	0.76385 (6)	0.94715 (5)	0.05124 (18)	
Cl3A	0.68978 (5)	0.70470 (5)	1.29886 (5)	0.0547 (2)	
Cl4A	0.33577 (4)	0.72377 (6)	1.10872 (5)	0.04826 (17)	
C11A	0.16642 (14)	0.78908 (17)	0.70221 (15)	0.0313 (5)	
C12A	0.10421 (16)	0.71386 (19)	0.67392 (17)	0.0386 (5)	
H12A	0.1136	0.6557	0.7079	0.046*	
C13A	0.02828 (16)	0.7229 (2)	0.59643 (17)	0.0419 (6)	
H13A	−0.0144	0.6717	0.5778	0.050*	
C14A	0.01600 (15)	0.8069 (2)	0.54725 (16)	0.0387 (5)	
C15A	0.07758 (16)	0.8833 (2)	0.57343 (17)	0.0425 (6)	
H15A	0.0686	0.9405	0.5384	0.051*	
C16A	0.15197 (15)	0.87407 (18)	0.65141 (16)	0.0369 (5)	
H16A	0.1937	0.9261	0.6706	0.044*	
C21A	0.45990 (15)	0.73872 (17)	1.03485 (15)	0.0332 (5)	
C22A	0.54822 (16)	0.74224 (18)	1.04103 (16)	0.0357 (5)	
C23A	0.61967 (16)	0.72962 (18)	1.12165 (18)	0.0405 (6)	
H23A	0.6796	0.7304	1.1248	0.049*	
C24A	0.60090 (17)	0.71594 (18)	1.19714 (17)	0.0401 (6)	
C25A	0.51466 (17)	0.71352 (18)	1.19431 (16)	0.0389 (5)	
H25A	0.5032	0.7042	1.2469	0.047*	
C26A	0.44472 (15)	0.72506 (17)	1.11274 (16)	0.0346 (5)	
O1W	0.23342 (15)	0.51628 (16)	0.73321 (13)	0.0524 (5)	
H1WA	0.250 (2)	0.487 (2)	0.7831 (12)	0.063*	
H1WB	0.1870 (15)	0.495 (2)	0.6911 (16)	0.063*	

### Atomic displacement parameters (Å^2^)

**Table:**

	*U*^11^	*U*^22^	*U*^33^	*U*^12^	*U*^13^	*U*^23^
C1	0.0239 (10)	0.0309 (11)	0.0321 (11)	0.0011 (8)	0.0063 (8)	−0.0005 (9)
O1	0.0247 (7)	0.0512 (10)	0.0287 (8)	0.0024 (7)	0.0073 (6)	0.0029 (7)
N1	0.0257 (9)	0.0420 (11)	0.0255 (9)	−0.0055 (8)	0.0054 (7)	0.0023 (8)
C2	0.0249 (10)	0.0349 (11)	0.0321 (11)	−0.0035 (9)	0.0045 (8)	−0.0004 (9)
N2	0.0203 (8)	0.0445 (11)	0.0294 (10)	−0.0007 (8)	0.0033 (7)	0.0021 (8)
S1	0.0294 (3)	0.0704 (5)	0.0407 (3)	−0.0145 (3)	0.0121 (3)	−0.0018 (3)
Cl1	0.0274 (3)	0.0557 (4)	0.0352 (3)	−0.0004 (2)	−0.0016 (2)	−0.0011 (3)
Cl2	0.0476 (4)	0.0498 (4)	0.0882 (6)	0.0034 (3)	0.0070 (4)	−0.0203 (4)
Cl3	0.0285 (3)	0.1409 (9)	0.0451 (4)	−0.0121 (4)	−0.0059 (3)	0.0145 (5)
Cl4	0.0482 (4)	0.0483 (4)	0.0538 (4)	0.0070 (3)	0.0156 (3)	−0.0073 (3)
C11	0.0243 (10)	0.0318 (11)	0.0283 (11)	−0.0001 (8)	0.0051 (8)	0.0008 (9)
C12	0.0244 (10)	0.0415 (12)	0.0309 (11)	−0.0046 (9)	0.0069 (8)	−0.0018 (10)
C13	0.0325 (11)	0.0442 (13)	0.0266 (11)	−0.0031 (10)	0.0074 (9)	−0.0009 (10)
C14	0.0266 (10)	0.0337 (11)	0.0328 (12)	0.0005 (9)	0.0022 (9)	0.0001 (9)
C15	0.0239 (10)	0.0450 (13)	0.0357 (12)	0.0045 (9)	0.0076 (9)	0.0004 (10)
C16	0.0266 (10)	0.0403 (12)	0.0310 (11)	0.0042 (9)	0.0091 (9)	0.0000 (9)
C21	0.0193 (9)	0.0481 (13)	0.0278 (11)	−0.0036 (9)	0.0030 (8)	0.0015 (10)
C22	0.0274 (11)	0.0459 (14)	0.0442 (14)	−0.0050 (10)	0.0088 (10)	−0.0036 (11)
C23	0.0320 (12)	0.0644 (18)	0.0400 (14)	−0.0158 (12)	0.0072 (10)	−0.0089 (13)
C24	0.0245 (11)	0.079 (2)	0.0371 (13)	−0.0095 (12)	0.0043 (10)	0.0078 (13)
C25	0.0254 (11)	0.0633 (17)	0.0447 (14)	0.0073 (11)	0.0078 (10)	0.0131 (13)
C26	0.0259 (10)	0.0467 (14)	0.0363 (12)	0.0020 (10)	0.0090 (9)	0.0027 (11)
C1A	0.0234 (10)	0.0387 (12)	0.0305 (11)	−0.0002 (9)	0.0084 (8)	0.0006 (9)
O1A	0.0302 (8)	0.0364 (9)	0.0341 (8)	−0.0032 (7)	0.0043 (6)	−0.0006 (7)
N1A	0.0277 (9)	0.0350 (10)	0.0300 (10)	−0.0005 (8)	0.0045 (7)	−0.0024 (8)
C2A	0.0239 (10)	0.0400 (12)	0.0288 (11)	−0.0017 (9)	0.0084 (8)	−0.0006 (9)
N2A	0.0297 (9)	0.0375 (11)	0.0272 (9)	−0.0005 (8)	0.0008 (8)	0.0005 (8)
S1A	0.0343 (3)	0.0375 (3)	0.0342 (3)	0.0004 (2)	0.0049 (2)	0.0024 (2)
Cl1A	0.0317 (3)	0.0725 (5)	0.0405 (3)	0.0089 (3)	−0.0041 (2)	0.0019 (3)
Cl2A	0.0459 (4)	0.0694 (5)	0.0421 (3)	−0.0076 (3)	0.0207 (3)	−0.0047 (3)
Cl3A	0.0466 (4)	0.0533 (4)	0.0410 (4)	0.0015 (3)	−0.0098 (3)	0.0064 (3)
Cl4A	0.0348 (3)	0.0623 (4)	0.0491 (4)	−0.0024 (3)	0.0173 (3)	0.0007 (3)
C11A	0.0233 (10)	0.0395 (12)	0.0289 (11)	0.0003 (9)	0.0073 (8)	−0.0008 (9)
C12A	0.0285 (11)	0.0437 (13)	0.0362 (12)	−0.0029 (10)	0.0037 (9)	0.0037 (10)
C13A	0.0290 (11)	0.0490 (14)	0.0396 (13)	−0.0052 (10)	0.0038 (10)	−0.0033 (11)
C14A	0.0254 (10)	0.0526 (15)	0.0326 (12)	0.0073 (10)	0.0049 (9)	−0.0016 (11)
C15A	0.0351 (12)	0.0481 (14)	0.0400 (13)	0.0070 (11)	0.0094 (10)	0.0071 (11)
C16A	0.0275 (10)	0.0403 (13)	0.0383 (12)	0.0005 (9)	0.0073 (9)	0.0012 (10)
C21A	0.0288 (11)	0.0338 (11)	0.0303 (11)	−0.0003 (9)	0.0035 (9)	−0.0011 (9)
C22A	0.0314 (11)	0.0377 (12)	0.0339 (12)	−0.0021 (9)	0.0077 (9)	−0.0026 (10)
C23A	0.0273 (11)	0.0412 (13)	0.0454 (14)	−0.0015 (9)	0.0052 (10)	−0.0031 (11)
C24A	0.0385 (13)	0.0328 (12)	0.0359 (12)	0.0004 (10)	−0.0009 (10)	0.0013 (10)
C25A	0.0428 (13)	0.0375 (13)	0.0311 (12)	−0.0013 (10)	0.0078 (10)	0.0023 (10)
C26A	0.0282 (11)	0.0373 (12)	0.0335 (12)	0.0003 (9)	0.0064 (9)	0.0008 (10)
O1W	0.0528 (12)	0.0601 (13)	0.0406 (11)	−0.0075 (10)	0.0133 (9)	−0.0067 (9)

### Geometric parameters (Å, °)

**Table:**

C1—O1	1.229 (3)	C1A—N1A	1.376 (3)
C1—N1	1.380 (3)	C1A—C11A	1.493 (3)
C1—C11	1.489 (3)	N1A—C2A	1.398 (3)
N1—C2	1.394 (3)	N1A—H1A	0.874 (10)
N1—H1	0.866 (10)	C2A—N2A	1.342 (3)
C2—N2	1.341 (3)	C2A—S1A	1.664 (2)
C2—S1	1.666 (2)	N2A—C21A	1.423 (3)
N2—C21	1.424 (3)	N2A—H2A	0.877 (10)
N2—H2	0.873 (10)	Cl1A—C14A	1.743 (2)
Cl1—C14	1.739 (2)	Cl2A—C22A	1.732 (3)
Cl2—C22	1.727 (3)	Cl3A—C24A	1.741 (2)
Cl3—C24	1.751 (3)	Cl4A—C26A	1.735 (2)
Cl4—C26	1.726 (3)	C11A—C12A	1.392 (3)
C11—C16	1.397 (3)	C11A—C16A	1.398 (3)
C11—C12	1.403 (3)	C12A—C13A	1.393 (3)
C12—C13	1.395 (3)	C12A—H12A	0.9500
C12—H12	0.9500	C13A—C14A	1.376 (4)
C13—C14	1.389 (3)	C13A—H13A	0.9500
C13—H13	0.9500	C14A—C15A	1.396 (4)
C14—C15	1.385 (3)	C15A—C16A	1.385 (3)
C15—C16	1.388 (3)	C15A—H15A	0.9500
C15—H15	0.9500	C16A—H16A	0.9500
C16—H16	0.9500	C21A—C26A	1.393 (3)
C21—C22	1.394 (4)	C21A—C22A	1.392 (3)
C21—C26	1.394 (3)	C22A—C23A	1.392 (3)
C22—C23	1.390 (4)	C23A—C24A	1.386 (4)
C23—C24	1.372 (4)	C23A—H23A	0.9500
C23—H23	0.9500	C24A—C25A	1.376 (4)
C24—C25	1.386 (4)	C25A—C26A	1.389 (3)
C25—C26	1.396 (3)	C25A—H25A	0.9500
C25—H25	0.9500	O1W—H1WA	0.855 (10)
C1A—O1A	1.234 (3)	O1W—H1WB	0.855 (10)
			
O1—C1—N1	121.66 (19)	O1A—C1A—C11A	120.9 (2)
O1—C1—C11	121.1 (2)	N1A—C1A—C11A	116.4 (2)
N1—C1—C11	117.2 (2)	C1A—N1A—C2A	128.1 (2)
C1—N1—C2	128.6 (2)	C1A—N1A—H1A	118.0 (19)
C1—N1—H1	116.8 (19)	C2A—N1A—H1A	113.9 (19)
C2—N1—H1	114.6 (19)	N2A—C2A—N1A	115.6 (2)
N2—C2—N1	115.6 (2)	N2A—C2A—S1A	123.96 (17)
N2—C2—S1	123.59 (16)	N1A—C2A—S1A	120.39 (17)
N1—C2—S1	120.80 (18)	C2A—N2A—C21A	121.5 (2)
C2—N2—C21	121.19 (19)	C2A—N2A—H2A	122 (2)
C2—N2—H2	121 (2)	C21A—N2A—H2A	116 (2)
C21—N2—H2	117 (2)	C12A—C11A—C16A	119.0 (2)
C16—C11—C12	119.2 (2)	C12A—C11A—C1A	123.9 (2)
C16—C11—C1	117.0 (2)	C16A—C11A—C1A	117.0 (2)
C12—C11—C1	123.8 (2)	C11A—C12A—C13A	120.8 (2)
C13—C12—C11	120.5 (2)	C11A—C12A—H12A	119.6
C13—C12—H12	119.8	C13A—C12A—H12A	119.6
C11—C12—H12	119.8	C14A—C13A—C12A	119.1 (2)
C14—C13—C12	118.9 (2)	C14A—C13A—H13A	120.5
C14—C13—H13	120.6	C12A—C13A—H13A	120.5
C12—C13—H13	120.6	C13A—C14A—C15A	121.5 (2)
C15—C14—C13	121.6 (2)	C13A—C14A—Cl1A	119.6 (2)
C15—C14—Cl1	119.17 (18)	C15A—C14A—Cl1A	118.9 (2)
C13—C14—Cl1	119.25 (19)	C16A—C15A—C14A	118.8 (2)
C14—C15—C16	119.3 (2)	C16A—C15A—H15A	120.6
C14—C15—H15	120.4	C14A—C15A—H15A	120.6
C16—C15—H15	120.4	C15A—C16A—C11A	120.8 (2)
C15—C16—C11	120.6 (2)	C15A—C16A—H16A	119.6
C15—C16—H16	119.7	C11A—C16A—H16A	119.6
C11—C16—H16	119.7	C26A—C21A—C22A	117.8 (2)
C22—C21—C26	117.9 (2)	C26A—C21A—N2A	121.7 (2)
C22—C21—N2	121.1 (2)	C22A—C21A—N2A	120.5 (2)
C26—C21—N2	121.0 (2)	C21A—C22A—C23A	121.6 (2)
C23—C22—C21	121.9 (3)	C21A—C22A—Cl2A	119.99 (18)
C23—C22—Cl2	118.9 (2)	C23A—C22A—Cl2A	118.4 (2)
C21—C22—Cl2	119.17 (18)	C24A—C23A—C22A	118.2 (2)
C24—C23—C22	118.0 (3)	C24A—C23A—H23A	120.9
C24—C23—H23	121.0	C22A—C23A—H23A	120.9
C22—C23—H23	121.0	C25A—C24A—C23A	122.1 (2)
C23—C24—C25	122.9 (2)	C25A—C24A—Cl3A	119.3 (2)
C23—C24—Cl3	118.6 (2)	C23A—C24A—Cl3A	118.6 (2)
C25—C24—Cl3	118.6 (2)	C24A—C25A—C26A	118.4 (2)
C24—C25—C26	117.8 (3)	C24A—C25A—H25A	120.8
C24—C25—H25	121.1	C26A—C25A—H25A	120.8
C26—C25—H25	121.1	C25A—C26A—C21A	121.8 (2)
C21—C26—C25	121.5 (2)	C25A—C26A—Cl4A	118.7 (2)
C21—C26—Cl4	119.26 (17)	C21A—C26A—Cl4A	119.46 (17)
C25—C26—Cl4	119.2 (2)	H1WA—O1W—H1WB	118 (3)
O1A—C1A—N1A	122.7 (2)		
			
O1—C1—N1—C2	−1.6 (4)	O1A—C1A—N1A—C2A	5.1 (4)
C11—C1—N1—C2	177.8 (2)	C11A—C1A—N1A—C2A	−174.6 (2)
C1—N1—C2—N2	−1.6 (4)	C1A—N1A—C2A—N2A	−1.7 (3)
C1—N1—C2—S1	178.25 (19)	C1A—N1A—C2A—S1A	178.52 (19)
N1—C2—N2—C21	−175.6 (2)	N1A—C2A—N2A—C21A	−179.3 (2)
S1—C2—N2—C21	4.6 (3)	S1A—C2A—N2A—C21A	0.4 (3)
O1—C1—C11—C16	−12.9 (3)	O1A—C1A—C11A—C12A	−155.0 (2)
N1—C1—C11—C16	167.7 (2)	N1A—C1A—C11A—C12A	24.7 (3)
O1—C1—C11—C12	165.2 (2)	O1A—C1A—C11A—C16A	21.6 (3)
N1—C1—C11—C12	−14.1 (3)	N1A—C1A—C11A—C16A	−158.7 (2)
C16—C11—C12—C13	1.0 (4)	C16A—C11A—C12A—C13A	−0.3 (4)
C1—C11—C12—C13	−177.1 (2)	C1A—C11A—C12A—C13A	176.2 (2)
C11—C12—C13—C14	0.0 (4)	C11A—C12A—C13A—C14A	0.9 (4)
C12—C13—C14—C15	−0.7 (4)	C12A—C13A—C14A—C15A	−0.3 (4)
C12—C13—C14—Cl1	178.12 (19)	C12A—C13A—C14A—Cl1A	179.9 (2)
C13—C14—C15—C16	0.3 (4)	C13A—C14A—C15A—C16A	−0.9 (4)
Cl1—C14—C15—C16	−178.50 (19)	Cl1A—C14A—C15A—C16A	179.0 (2)
C14—C15—C16—C11	0.7 (4)	C14A—C15A—C16A—C11A	1.4 (4)
C12—C11—C16—C15	−1.4 (4)	C12A—C11A—C16A—C15A	−0.8 (4)
C1—C11—C16—C15	176.9 (2)	C1A—C11A—C16A—C15A	−177.6 (2)
C2—N2—C21—C22	−100.5 (3)	C2A—N2A—C21A—C26A	−85.7 (3)
C2—N2—C21—C26	81.6 (3)	C2A—N2A—C21A—C22A	95.8 (3)
C26—C21—C22—C23	−1.8 (4)	C26A—C21A—C22A—C23A	2.0 (4)
N2—C21—C22—C23	−179.8 (2)	N2A—C21A—C22A—C23A	−179.5 (2)
C26—C21—C22—Cl2	177.95 (19)	C26A—C21A—C22A—Cl2A	−177.46 (18)
N2—C21—C22—Cl2	0.0 (3)	N2A—C21A—C22A—Cl2A	1.1 (3)
C21—C22—C23—C24	0.7 (4)	C21A—C22A—C23A—C24A	−1.8 (4)
Cl2—C22—C23—C24	−179.1 (2)	Cl2A—C22A—C23A—C24A	177.66 (19)
C22—C23—C24—C25	0.2 (4)	C22A—C23A—C24A—C25A	0.7 (4)
C22—C23—C24—Cl3	179.5 (2)	C22A—C23A—C24A—Cl3A	−177.60 (19)
C23—C24—C25—C26	0.1 (4)	C23A—C24A—C25A—C26A	0.1 (4)
Cl3—C24—C25—C26	−179.2 (2)	Cl3A—C24A—C25A—C26A	178.41 (19)
C22—C21—C26—C25	2.1 (4)	C24A—C25A—C26A—C21A	0.1 (4)
N2—C21—C26—C25	−179.9 (2)	C24A—C25A—C26A—Cl4A	−179.14 (19)
C22—C21—C26—Cl4	−176.13 (19)	C22A—C21A—C26A—C25A	−1.1 (4)
N2—C21—C26—Cl4	1.8 (3)	N2A—C21A—C26A—C25A	−179.6 (2)
C24—C25—C26—C21	−1.3 (4)	C22A—C21A—C26A—Cl4A	178.12 (18)
C24—C25—C26—Cl4	176.9 (2)	N2A—C21A—C26A—Cl4A	−0.4 (3)

### Hydrogen-bond geometry (Å, °)

**Table:**

*D*—H···*A*	*D*—H	H···*A*	*D*···*A*	*D*—H···*A*
N1—H1···O1W	0.87 (1)	2.21 (2)	2.997 (3)	151 (3)
N2—H2···O1	0.87 (1)	1.97 (2)	2.627 (2)	131 (3)
N2—H2···O1A^i^	0.87 (1)	2.26 (2)	2.931 (3)	133 (2)
N1A—H1A···O1W	0.87 (1)	1.96 (1)	2.816 (3)	164 (3)
N2A—H2A···O1A	0.88 (1)	1.98 (3)	2.637 (3)	130 (3)
N2A—H2A···O1^ii^	0.88 (1)	2.31 (2)	3.001 (3)	136 (3)
O1W—H1WA···S1A	0.86 (1)	2.67 (3)	3.215 (2)	123 (3)
O1W—H1WA···Cl3^iii^	0.86 (1)	2.84 (3)	3.388 (2)	123 (3)
O1W—H1WB···S1	0.86 (1)	2.36 (2)	3.091 (2)	144 (3)

Symmetry codes: (i) *x*, −*y*+3/2, *z*−1/2; (ii) *x*, −*y*+3/2, *z*+1/2; (iii) −*x*, −*y*+1, −*z*+1.
